# Knowledge, practice, and associated factors of preoperative patient teaching among surgical unit nurses, at Northwest Amhara Comprehensive Specialized Referral Hospitals, Northwest Ethiopia, 2022

**DOI:** 10.1186/s12912-023-01175-2

**Published:** 2023-01-21

**Authors:** Astewil Moges Bazezew, Nurhusen Nuru, Tizta Gebeyehu Demssie, Desalegn Getachew Ayele

**Affiliations:** Department of Surgical Nursing, School of Nursing, College of Medicine and Health Science, University of Gondar, Gondar, Ethiopia

**Keywords:** Knowledge, Practice, And preoperative teaching

## Abstract

**Background:**

Preoperative teaching practice is very important to surgical clients in freeing them from anxiety and post-operative complications. The preoperative education received by the patients depends on the knowledge and experience of nurses. The diversity in the degree of knowledge and experience possessed by nurses may result in inadequate and ineffective preoperative preparation of patients. Therefore, this study aimed to assess the knowledge, practice, and associated factors of preoperative patient teaching among nurses working at surgical units in Northwest Amhara Comprehensive Specialized Referral Hospitals, Northwest Ethiopia, 2022.

**Method:**

An institutional-based cross-sectional study triangulated with a qualitative approach was conducted from April to June 2022. The data were collected using a semi-structured self-administered questionnaire and in-depth interviews. The descriptive statistics were presented in text and tables. Analytical analysis schemes including bivariable and multivariable logistic regression were computed considering *P*-value < 0.05 to identify statistically significant factors. Qualitative data were analyzed with thematic analysis.

**Result:**

A total of 406 participants were involved in this study with a 95.8% response rate. The adequate knowledge of nurses was 61.6% with 95% CI: (56.7, 66.3) and significantly associated with being male, nurse use of guidelines, nurses they have been trained, and nurses’ who say they do not a staff shortage had good knowledge than the counterparts. Good practice of nurses regarding preoperative patient education was 46.3% with 95% CI (41.4, 51.0) significantly associated with the presence of preoperative teaching guidelines, took training on patient education, nurses who said no staff shortage, and knowledge of preoperative patient education.. Nurses have a positive interaction with the patient and much work experience had good practice but lack of training; inadequate supplies and lack of professional prerequisites are some of the barriers identified.

**Conclusion:**

Nurses’ knowledge and practice regarding preoperative patient teaching were found to be inadequate. So, it is better to strengthen training, adequate staffing, equip wards with standardized guidelines and teaching materials, motivate and create a safe working environment. Most nurses explore factors of preoperative patients’ teaching as institutional, Nurse’s related, and patient-related factors.

## Background

Preoperative teaching is an intervention conducted before surgery that aims to improve patients’ knowledge, health behaviors, and outcomes [1]. Providing adequate and timely pre-operative education is critical, ineffective education can lead to higher anxiety, a higher risk of problems, longer hospital stays, and more readmissions [2].

Surgical morbidity is a significant health issue worldwide. It is estimated that > 230 million surgical procedures are performed each year [3]. In Ethiopia, there were 20 (2.49%) deaths described at 24 h and 26 (3.29%) deaths recorded at 48 h after surgery and anesthesia [4]. The perioperative period is stressful, with many pathophysiologic alterations rendering patients vulnerable to several potential adverse events [5]. Research suggests that preoperative teaching enhances patients’ recovery from surgery [6].

The preoperative education received by the patients depends on the knowledge and experience of nurses [7]. The diversity in the degree of knowledge and experience possessed by nurses may result in inadequate and ineffective preoperative preparation of patients [5].

Perioperative nurses should have the requisite knowledge and skill to assess, diagnose, plan, intervene, and evaluate the outcomes of interventions. Before surgery, a perioperative nurse must assess and prepare surgical patients by addressing their physiological, spiritual, and psychological reactions toward surgery [8].

Surgical education is frequently provided in the postoperative room while the patient is sedated and recovering from anesthesia, but many patients want to learn more before the procedure (preoperative teaching) [9]. Preoperative patient education is a key part of nursing consideration aimed at helping patients to clarify information about their operation, and what happens after surgery, based on patient need, level of knowledge, and patient condition [10].

It has proven difficult to develop formal preoperative teaching programs for nurses in perioperative settings [11]. Although nurses act as key educators in patient teaching, little is known about their current practice in the provision of such teaching for surgical patients [12].

Despite the universally acknowledged importance of preoperative teaching, its implementation especially in developing countries is low, this is largely detailed to be associated with unawareness, inadequate knowledge among staff nurses on the concept of preoperative teaching, nurses‟ shortage in the hospitals, lack of time, and work overload are factors [13]. Therefore, the purpose of this study is to assess the knowledge, practice, and determinant factors of preoperative patient teaching among nurses in Northwest Amhara Regional State Referral Hospitals, Northwest Ethiopia.

## Methods and materials

### Study design, area and period

An institutional-based cross-sectional study design, mixed-method was conducted April to June /2022G.c at Northwest Amhara comprehensive specialized referral Hospitals, Northwest Ethiopia. Amhara region is one of the regions in Ethiopia and is located in the Northwest part of Ethiopia. Its Capital city is Bahir Dar, which is located 565 km from Addis Ababa, the capital city of Ethiopia. There are five government referral hospitals like the University of Gondar Comprehensive Specialized Referral Hospital (UoGCSRH), Felegehiwot Comprehensive Specialized Referral Hospital (FHRH), Tibebegion Comprehensive Specialized Hospital (TGCSH), Debre Markos Comprehensive Specialized (DMCSH), and Debre Tabor Comprehensive Specialized Hospital (DTCSH). All hospitals provide outpatient and inpatient services for more than 22,000,000 million people living in their catchment area. Those hospitals have currently 1682 nurses working and the total number of nurses who are working in an emergency including trauma unit, operation room, recovery ward, surgical and orthopedics ward, and surgical ICU is 745 [4, 14–17].

### Source population and study population

#### Source population

Nurses who had been working in Northwest Comprehensive State Referral Hospitals, Northwest Ethiopia,2022.

#### Study Population

All nurses who had been working in selected units or wards (surgical emergency, operation room, and recovery ward, surgical and orthopedics ward, and surgical ICU ward) at UoGCSRH, DTRH, DMRH, TGRH, and FHRH during the data collection period, 2022.

### Inclusion and Exclusion criteria

#### Inclusion criteria

All nurses who were working in the surgical emergency, operating theatre room, recovery ward, surgical ICU, surgical & orthopedic ward of Northwest Amhara comprehensive Referral Hospitals, Northwest Ethiopia during the time of data collection.

#### Exclusion criteria

Nurses’ managers who were working in the selected area were excluded.

### Sample size determination and sampling technique

#### Sample size determination

The sample size was determined by using single population proportion formula using 95% confidence level (Z = 1.96), degree of precision (marginal error) = 5%, and proportion (*p* = 50% $n=\frac{{z}^{2}*p(1-p)}{{d}^{2}}$

Where; *n* = the required sample size.

*P* = proportion of nursing students; 50% = 0.5 (since there was no previous study in Ethiopia).

z = degree of accuracy at 95% = 1.96.

d = margin of error = 0.05

$$n=\frac{({1.96)}^{2}*0.5(1-0.5)}{{(0.05)}^{2}}$$

None response 38.4≈ 39(10%). The final sample size was 423.

#### Sampling technique

A stratified sampling technique was employed to recruit the required participant for the study. First, the study participants are stratified from each hospital and working ward/unit and allocated the required sample for each stratum proportionally. Finally, a proportional number of participants were selected by a simple random sampling method. According to all hospitals of human resources, and administration on reports the total number of nurses who were working in this unit or wards was 745. Based on the number of nurses working in each hospital, 423 samples were proportionally allocated from the 745. Finally, those participants had been taken by a simple random sampling technique.

### Operational definitions

Preoperative teaching: is a process of educating surgical clients before undergoing a surgical operation [18], Which aims to improve patients knowledge, health behavior and health outcome related to the surgical procedures [19].

Good knowledge: If the study participants answer the knowledge questions above or equal to the computed median [10] were considered as having good knowledge.

Poor knowledge: If the study participants answer, the knowledge questions below the computed median [10] were considered as having poor knowledge.

Good practice: The study participants who answer above or equal to the computed median [17] of practice questions were considered as having good practice.

Poor practice: The study participants who answer below the computed median [17] of practice questions were considered as having poor practice.

Hospital support: Organizational support in completing nursing duties to allow time for patient teaching [20].

The staff adequacy: The variable used in this study was a measure of perceived adequacy of staffing on participants’ units [21].

Tight operation schedule: The variable used in this study was a measure of perceived low priority on patient teaching when they were faced with issues of time and heavy workloads [12].

### Data collection tool and procedure

Data was collected by using a self-administered questionnaire (which involves 15 questions for Socio-demographic, institutional, and other factors.16 questions for knowledge. and 14 questions for practice. The tool was adapted from different literature and association perioperative registered nurses [12, 22–30]. The questionnaires are prepared in the English language based on the study objectives, focusing on the background information of preoperative patient education and practice.

Five BSc Nurse who are working other than the study area are recruited for data collection and two MSc holder nurses are recruited as a supervisor Overall, the data collection process was coordinated and supervised by the principal investigator.

### Data quality assurance

To ensure the quality of data one-day training was given to data collectors and supervisors regarding the structured questionnaire (on the objective of the study and how to collect the data). A week before starting the actual period of data collection was a pretest on 5% of the sample at Woldia Referral hospital. Regular supervision was done to check the consistency and completeness of the filled-out questionnaires, by the supervisors and principal investigator. Face validity was checked by experts and advisors. By using SPSS version 20, Cronbach’s alpha was calculated to test the internal consistency of items. The tests showed 0.775 and 0.941 for knowledge and practice. After the actual data collection process, the collected data were cross-checked for questionnaires’ consistency and completeness. During the interview, the investigator encouraged and probed participants to provide their opinion about factors of preoperative education. The investigator tried to keep a neutral position and encourage and probe participants to provide their opinion about factors of preoperative patient teaching. Credibility was maintained by persistent engagement and presentation of preliminary findings to colleagues of the investigator through email. Transferability was maintained by using purposive sampling and to manage conformability and dependability there was no relationship between the investigator and study participants, during the interview the investigator tried to keep a neutral position.

### Data processing and analyzing

All the questionnaires were checked visually and coded and the data were entered into Epi Info version 7 and exported into SPSS version 20 for analysis. Frequencies, percentages, and median with IQR were computed to describe the key variables of the study. Binary logistic regression was run to determine significant relations of independent variables with the dependent variable and all independent variables, which are less than 0.2 in bivariate analysis, were entered into multivariable logistic regressions. A *P*-value of < 0.05 was considered significant for all analyses. Binary logistic regression analysis was carried out to identify the independent factors related to the outcome variable.

## Results

### Sociodemographic characteristics of the study participants

A total of 423, with a response rate of 95.8% (406) of study participants were involved in this study. Among respondents 228 (56.2%) were males. In this study 209 (51.5%) of the age group was between 28–34 and 229 (56.4%) were married. Out of 406 nurses, most of the respondents (85.7%) had a bachelor's degree, and two hundred twenty-two 223 (54.9%) of the respondents had < 5 years of work experience. Two hundred sixteen 216 (53.2%) of participants' monthly salary ranges from 7001–9000 (Table 1).

**Table 1:** Sociodemographic characteristics of the study participants on preoperative patient teaching among surgical unit nurses in Northwest Amhara Comprehensive Specialized Referral Hospitals, Northwest Ethiopia, 2022(*N* = 406)

Variables	Response	Frequency (*N* = 423)	Percentage (%)
Sex	Male	228	56.2
	Female	178	43.8
Age	≤ 27	113	27.8
	28–34	209	51.5
	≥ 35	84	20.7
Marital status	Single	171	42.4
	Married	229	56.1
	Divorced	6	1.5
Educational Status	Diploma	27	6.7
	Degree	336	82.8
	Masters	43	10.6
Work Experience	≤ 5 years	223	54.9
	6–10	134	33.0
	11–15	35	8.6
	≥ 16	14	3.4
Monthly Salary (ETB)	≤ 5000	31	7.6
	5001–7000	103	25.4
	7001–9000	216	53.2
	≥ 9000	56	13.8

### Institutional-related and other factors

Nurses did not take training about preoperative patient teaching 294(72.4%) from 406 respondents. 218(53.7%) of the respondents reports that they were a staff shortage, and only 104(25.6%) of participants have notified the presence of guidelines or protocols in their working area. Out of the study participants, 281 (69.2%) and 213 (52.5%) were having a tight operation schedule and no hospital supports respectively. Of 406 study participants, nearly more than half of 284 (70%) participants offered that there are no language barriers (Table 2).

**Table 2:** Institutional and other related factors of the study participants on preoperative patient teaching among surgical unit nurses in Northwest Amhara Comprehensive Specialized Referral Hospitals, Northwest Ethiopia, 2022 (*N* = 406)

Variables	Response	Frequency (*N* = 423)	Percentage (%)
Working place	UoGCSH	92	27.7
	FHCSH	128	31.5
	TGCSH	86	21.2
	DTCSH	37	9.1
	DMCSH	63	15.5
Ward	OR	110	27.1
	Surgical ward	93	22.9
	Orthopedic ward	35	8.6
	Emergency	75	18.5
	Recovery	37	9.1
	ICU	56	13.8
Daily working hours	≤ 8 h	259	63.8
	≥ 9 h	147	36.2
Training on preoperative patient teaching	Yes	112	27.6
	No	294	72.4
Presence of guidelines	Yes	104	25.6
	No	302	74.4
Staff shortage	Yes	188	46.3
	No	218	53.7
Tight operation schedule	Yes	281	69.2
	No	125	30.8
Language barrier	Yes	122	30
	No	284	70
Hospital supports	Yes	193	47.5
	No	213	52.5

### Knowledge of nurses on preoperative patient teaching

The overall median knowledge score of the study participants on preoperative patient teaching was 10. In this study, 250 (61.6%) with 95% CI: (56.7, 66.3) of the participants had good knowledge. Among a total of knowledge assessment questions, 287(70.7%) of participants correctly answered the statement of preoperative information should not be limited to surgical patients but involves their families and 344(84.7%) of the participants correctly answered before surgery perioperative nurse should schedule preoperative education. 218 (53.7%) of the participants gave the correct answer of preoperative education increases preoperative anxiety for a patient undergoing surgery. Only 141(34.7%) of the participants were correctly respond Preoperative instruction assists surgical patients in the preoperative period only (Table 3).

**Table 3:** Nurse’s responses on knowledge of preoperative patient teaching in Northwest Amhara Comprehensive Specialized Referral Hospitals, Northwest Ethiopia 2022 (*N* = 406)

Statement about preoperative patient teaching	Yes/No	Correct Answer		Wrong Answer	
General knowledge		N	%	N	%
Preoperative information should not be limited to surgical patients but involves their families	Yes	287	70.7	119	29.3
Before surgery, perioperative nurses should schedule preoperative education	Yes	344	84.7	62	15.3
Preoperative teaching is the responsibility of nurses working in surgical units	Yes	271	66.7	135	33.3
Preoperative patient education increases preoperative anxiety in patients undergoing surgery	No	218	53.7	188	46.3
Preoperative patient education will minimize postoperative problems in surgical patients	Yes	301	74.1	105	25.9
Preoperative patient education helps patients understand what to expect following surgery	Yes	298	73.4	108	26.6
Preoperative instruction assists surgical patients in the preoperative period only	No	141	34.7	265	65.3
Teaching a patient about fasting times before to surgery is one type of preoperative instruction	Yes	303	74.6	103	25.4
Preoperative patient teaching includes medications to use perioperatively	Yes	288	70.9	118	29.1
Preoperative patient education involves explanations of the different forms of anesthetic to be used during surgery	Yes	228	56.2	178	43.8
Preoperative teaching includes information about the perioperative environment	Yes	291	71.7	115	28.3
Post-operative pain management is a part of preoperative teaching	Yes	205	50.5	201	49.5
Preoperative patients’ teaching involves postoperative deep breathing and coughing	Yes	263	64.8	143	35.2
Preoperative patient education involves early mobility and ambulation of postoperative patients	Yes	264	65	142	35
Preoperative patients’ education involves information about skin decontamination on the day of surgery	Yes	295	72.7	111	27.3
Preoperative patient education should advise patients undergoing surgery to keep their personal belongings to themselves	Yes	186	45.8	220	54.2

### Practice of nurses on preoperative patient teaching

The overall median practice score of the study participants on preoperative patient teaching was 17. In this study, 218 (53.7%) with 95% CI: (48.8, 58.1) of the participants had poor practice. Among practice assessment questions, 234(28%) of the respondents teach patients undergoing surgery within a specific time before surgery, and 164(40.4%) of the respondents always taught patients about fasting time before undergoing surgery. the respondents 168(40.6%), 178(43.8%), and 167(41.1%) were sometimes taught to patients undergoing surgery about skin hygiene on the day of surgery, instruct preoperative patients about postoperative deep breathing and coughing, and explain to the patient before undergoing surgery about the management of postoperative pain respectively. Among 406 participants, 168(40.6%) respondents reported nurses’ use of teaching materials when providing preoperative patient teaching (Table 4).

**Table 4:** Nurse’s responses on the practice of preoperative patient teaching in Northwest Amhara Comprehensive Specialized Referral Hospitals, Northwest Ethiopia 2022 (*N* = 406)

Preoperative patient teaching	Never		Sometimes		Always	
	No	%	No	%	No	%
Do you teach patients undergoing surgery within a specific time before surgery	56	13.8	116	28.6	234	28
Do you teach your patients about fasting time before undergoing surgery	76	18.7	166	40.9	164	40.4
Do you educate your patients about perioperative medications	81	20	173	42.6	152	37.4
Do you explain to your patients the types of anesthesia to use during surgery	131	32.3	165	40.6	110	27.1
Do you teach your patients regarding the perioperative environment to patients before surgery	92	22.7	159	39.2	155	38.2
Do you explain to the patient before undergoing surgery about the management of post-operative pain	119	29.3	167	41.1	120	29.6
Do you teach patients undergoing surgery about skin hygiene on the day of surgery	85	20.9	168	41.4	153	37.7
Do you teach your patients undergoing surgery that should leave valuables and remove all jewelry such as dentures, glasses, contact lenses, prostheses, makeup, nail polish, hairpins, or hairpiece before going into the operating room for surgery	42	10.3	186	45.8	177	43.6
Do you teach your patients differently than you teach their family members	138	34	165	40.6	103	25.4
Do you use teaching materials when providing preoperative patient teaching	165	40.6	151	37.2	90	22.2
Do you inform surgical patients about postoperative activity restrictions	116	28.6	157	38.7	133	32.8
Do you instruct preoperative patients about postoperative deep breathing and coughing	107	26.4	178	43.8	121	29.8
Do you verify the preparedness of patients before surgery	101	24.9	135	33.7	170	41.9
Do you keep a recording of the education	145	35.7	143	35.2	118	29.1

### Factors associated with the level of knowledge on preoperative patient teaching

In the binary logistic regression, seven from fifteen variables were found to have a significant association with participants’ level of knowledge on preoperative patient teaching at a *p*-value of < 0.2. However, after controlling for the effects of potentially confounding variables using multivariate logistic regression sex, nurses took training, nurses use guidelines and nurses said no staff shortage was found to be significant predictor for knowledge on preoperative patient education.

In this study, being male had 2.016 times more knowledge [AOR = 2.016, 95% CI (1.280–3.176)]. Also, nurses who took training on preoperative patient education and nurses use guidelines had 3.360 and 1.937 times more knowledge [AOR = 3.360; 95% CI (1.901–5.937), AOR = 1.937 95% CI (1.108–3.385)] respectively. Nurses said no staff shortage in their working unit had 1.96 times more knowledge [AOR = 1.960, 95% CI (1.239–3.100). (Table 5).

**Table 5:** Variable and multivariable analysis of factors associated with knowledge of nurses on preoperative patient teaching in Northwest Amhara Comprehensive Specialized Hospitals, Northwest Ethiopia 2022 (406)

Variables	Knowledge of nurses		COR (95%CI)	AOR (95%CI)	P-Value
	Good	Poor			
Sex					
Female	95	83	1	1	
Male	155	73	1.855(1.237–2.782)	2.016(1.280–3.176)	.002^**^
Educational status					
Diploma	10	17	1	1	
Degree	205	131	2.660(1.182–5.988)	2.164(.828–5.657)	.115
Masters	35	8	7.437(2.487–22.242)	2.802(.813–9.662)	.103
Work experience					
≤ 5 years	122	101	1	1	
6–10	88	46	1.584(1.016–2.468)	1.054(.618–1.797)	.847
11–15	28	7	3.311(1.388–7.898)	2.493(.952–6.526)	.063
≥ 16	12	2	4.967(4.086–22.711)	1.632(.297–8.958)	.573
Monthly salary					
≤ 500	17	14	1	1	
5001–7000	50	53	.777(.347–1.739)	.458(.176–1.188)	.108
7001–9000	134	82	1.346(.630–2.874)	.953(.379–2.393)	.918
≥ 9000	49	7	5.765(1.994–16.6770	2.906(.839–10.059)	.092
Staff shortage					
Yes	100	88	1	1	
No	150	68	1.941(1.294–2.911)	1.960(1.239–3.100)	.004**
Having a guideline					
No	172	130	1	1	
Yes	78	26	2.267(1.377–3.374)	1.937(1.108–3.385)	.020^**^
Took training					
No	160	134	1	1	
Yes	90	22	3.426(2.038–5.760)	3.360(1.901–5.937)	.000^**^

Variables show that significant association during multivariable logistic regression at**

*P*-value < 0.05 1 = reference

### Factors associated with practice the of nurses on preoperative patient education

In bivariate logistic regression analysis, ten from sixteen variables were found to have significant predictors at a *p*-value < 0.2. But after controlling for the effects of potentially confounding variables using multivariate logistic regression only nurses had guidelines, took training, and knowledge of nurses were found to be significant predictors for the practice of nurses on preoperative patient education at (*P* < 0.05).

Nurses who had guidelines on preoperative patient education were 1.919 times more likely to have good practice [AOR = 1.919; 95% CI (1.148–3.207)]. Nurses who took training on patient education were 2.049 times and nurses who said no staff shortage had 1.846 times more likely to have good practices for preoperative patient education [AOR = 2.049; 95% CI (1.227–3.420), AOR = 1.846; 95% CI (1.175–2.897)] respectively. The other significant variable is nurses who had knowledge of preoperative patient education were 3.276 times higher to have a good level of practice [AOR 3.276; 95% CI (2.022–5.309)] (Table 6).

**Table 6:** Bivariable and multivariable analysis of factors associated with the practice of nurses on preoperative patient teaching in Northwest Amhara Comprehensive Specialized Hospitals, Northwest Ethiopia 2022 (406)

Variables	Practice of nurses		COR (95%CI)	AOR (95%CI)	P –Value
	Good	Poor			
Educational status					
Diploma	11	16	1	1	
Degree	151	185	1.187(.535–2.635)	.665(.269–1.645)	.377
Masters	26	17	2.225(.834–5.935)	.811(.262–2.505)	.715
Language barrier					
Yes	63	59	1	1	
No	125	159	0.736(.481–1.27)	0.723(.442–1.182)	.578
Working experience					
≤ 5 years	94	129	1	1	
6–10	63	71	1.218(.791–1.874)	1.073(.662–1.740)	.774
11–15	23	12	2.630(1.246–5.551)	2.290(.987–5.314)	.054
≥ 16	8	6	1.830(.614–5.450)	1.367(.407–4.593)	.613
Staff shortage					
Yes	69	119	1	1	
No	119	99	2.073(1.391–3.089)	1.846(1.175–2.897)	.008^*^
Tight operation					
No	64	61	1	1	
Yes	124	157	1.328(0.871–2.027)	1.515(0.935–2.456)	.091
Guidelines					
No	123	179	1	1	
Yes	65	39	2.425(1.533–3.837)	1.919(1.148–3.207)	.013^**^
Took training					
No	114	180	1	1	
Yes	74	38	3.075(1.949–4.852)	2.049(1.227–3.420)	.006^**^
Hospital support					
No	92	121	1	1	
Yes	96	97	1.302(.880–1.925)	1.163(.744–1.819)	.508
Knowledge					
Poor knowledge	40	116	1	1	
Good knowledge	148	102	4.208(2.713–6.527)	3.276(2.022–5.309)	.000^**^

Variables that show significant association during multivariable logistic regression at ** *p*-value < 0.05 Reference = 1

A total of 13 nurses working in surgical units in three referral hospitals (Felegehiwot, Gondar and Tibebe Ghione) of Northwest Amhara were invited to take part in the study and 11 of these agreed to participate.

The analysis recognized two main themes and four sub-themes under the main themes related to barriers of nurses to patient education before surgery and facilitators. (Table 7).

**Table7:** Main themes with corresponding sub-themes in the content analysis

Main themes	Sub-themes
Barriers to preoperative patient education	Organizational factors
	Health care provider factors,
	Patient-related factors
Facilitators	Patient initiated factors

#### Barriers of nurses to preoperative patient education

These themes emerged from the nurses’ descriptions of challenges and limitations that participants faced when working in surgical units. This is described by the three sub-themes; organizational factors, health care provider factors, and patient-related factors.

##### Organizational factors

Nurses stated that their preoperative patient education is affected by organizational factors. Seven nurses expressed that organizational factor affects the delivery of preoperative patient education;

All participants described that limitations and inadequacy they encountered in their everyday experience were mainly related to lack of training, lack of guidelines, and lack of recognition from the hospital. One nurse stated: “*Many times they only offer work for the nurse, but they have forgotten need of something to improve the quality of patient education. Like: lack of training, absence of patient teaching schedules, not getting attention from the authorities and guidelines.”* (Nurse3).

*Even if you are standing up and teaching, when the boss sees you, he thinks that you are not doing work, but standing up and joking, so this is one of the things that keep me from teaching much*. (Nurse1).

Since there is no instruction to teach the patient in the facility, every professional wants to teach his ideas. “*Let me tell you about a case in which a man had an operation written on a card to be an NPO. Then I told him not to eat and I taught him why he should not eat. But a little later the doctors came and order him to eat. Then the man becomes confused and said you told me not to eat but he orders me to eat”.* (Nurse7).

*“It is important to know the procedure used to teach the patient, it is good idea to have at least some guidelines for each procedure. There is a big gap in this area. Because, I think education change a lot*. (Nurse8).

*“For example, the lack of a guideline makes you uneducated and unwilling to give an education. Next, when there was a lot of workloads, especially during the war, there was a lot of workloads and it was difficult not only to teach but also to give care”* (Nurse5).

Participants expressed those factors they encounter were a barrier to providing adequate preoperative patient education or inadequacy of fulfilling their perceived responsibilities. It is expressed as: *“it is uncommon for someone is encourage the patients to learn through the hospital, for example it would be nice if there was a program where all the nurses would take turns teaching patients”* (Nurse8).

Along with this, participants stated that the higher number of the patient, the harder it is to teach. ^*“*^*Sometimes it is difficult to provide education even when there are too many patients”.* (Nurse6).

A participant expressed that lack of training in the institution also factors for nurses: *“…the lack of different training is one of the reasons; in particular the hospital has never paid attention to such things. It is a big challenge for us…”* (Nurse6).

Most of the participants in this study expressed overwork as a reason for not teaching before the operation. “*In particular, we would like to hand over the responsibility to doctors and anesthesiologists due to the overcrowding and workload in the hospital”.* (Nurse3).

##### Health care provider’s factor

The low attitude of healthcare professionals toward their responsibility was reported to affect preoperative patient education. Most of the participants see the responsible of providing pre-operative education as the responsibility of physicians and anesthesiologists. ^*“*^*For my part I don’t think it was the responsibility of the nurse to see that the lesson was often given by doctors, but I did not pay attention to it because I said they teach”.* (Nurse2).

Participants expressed that those nurses who have worked for money years teach better in terms of experience. *“For example, I have seen a man who has worked in the ward for twenty years and I have seen him spend a lot of time teaching patients”.* (Nurse7).

“If you have worked for many years, you will treat all patients as family. I often see them getting closer and better. For example, I have worked for 7 years and my ability to communicate with a patient has changed dramatically. If so, it will help you to teach.” (Nurse1).

Most of the participants cited a lack of motivation in the patient teaching as a problem*.*^*”*^* Most of us only want to talk about what the patient is asking us to do or teach*^*”*^*.* (Nurse1).

In general, the concerns raised by the nurses were addressed by the majority of the participants as a lack of attention, a lack of compliance, a lack of cooperation with the hospital, a high number of patients, and a lack of knowledge. *“The problem I see with nurses is 1*^*st*^* negligence. You often have to teach, I mostly neglect it because of work pressures or because of other reasons. Again, I do not consider it necessary for nurses to teach patients. I think they should be taught by doctors or anesthesiologists. And I don’t teach when I am busy, not just me, because no one asks you whether you teach or not”.* (Nurse8).

##### Patient-related factors

Most of the participants reported that the patient’s health, as well as his or her educational status and general health condition, would be a barrier to pre-operative patient education.

“Well, I don’t think there is enough education for patients before the operation, but there are many reasons for this. For example, if the patient’s condition is complex, you will not be able to talk about education because you will not able to do anything if the patient is suffering from an illness or is incapable of teaching, so you will not able to teach”. (Nurse1).

“Another is that patient’s attitude toward nurses is declining. They want someone better than you, especially if their education is somewhat good and if they are a city dweller”. (Nurse6).

On the other hand, some patients may have a variety of complications when they need to be operated on. For example, *“if they have diabetes or high blood pressure or other related problems, the operation will be canceled. So, it can be difficult to teach. On the other hand, the community’s perception of the operation for example, the misconception that the lights will go out during the operation will increase patients fear. This will make it difficult to teach”*. (Nurse2).

“If an emergency surgery is needed, it is difficult not only not to teach the patient about the operation, but also to prepare it well for the operation, so things like this can happen and you will not be able to teach.” (Nurse4).

Two participants also raised language as a problem in teaching patients. *“Most of the patients were soldiers, especially during the war, and most of the patients from Oromia and Debub regions had difficulty communicating”.* (Nurse7).

#### Facilitators of preoperative patient education

##### Patient-initiated factors

Many participants feel that the good relationship between nurses and patients makes it easier for patients to ask questions, and that they have a good chance of teaching if nurses have a good knowledge of the operation. *“When the patient asks me, I share what I know well. Your relationship with the patient depends on what motivates you to teach the patient. When you have a good relationship, you have the freedom to say what you want”.* (Nurse9).

Providing enough human resources and teaching guidelines, running comprehensive educational programs, improving communication and good relationship with patients, and the hospital should support nurses and provide education to patients before the operation, and by removing existing difficulties and providing required knowledge through training, health care professionals will improve preoperative patient education and the quality of care by decreasing adverse events as described by respondents.

## Discussion

The purpose of study aimed to assess the knowledge, practice, and associated factors of preoperative patient teaching among nurses working at surgical units and the result of this study showed that the overall good knowledge of nurses on preoperative patient teaching was (61.6%) with 95% CI: (56.7, 66.3). This finding is consistent with a study in Nineveh, 62.5% [31]. Even though there is a difference in socioeconomic status and level of health sector development, the possible reason for the similarity between the current study and the study in Nineveh, Iraq might be using a similar study population (staff nurse), and study design.

This finding is lower than previous studies conducted in Kigali hospital, Rwanda (93%) [32]. The difference might be the study setting, the study population which was only operating theater nurses were involved in the study and this is because the OR is an environment that differs from other clinical areas. While nurses in other settings focus their attention on direct patient care and planning, OR nurses have a brief yet important period for patient care before surgery (anesthesia), with most time spent facilitating surgery [20]. And it might be small sample size (70) of nurses in the study while in the current study a total of 406 participants were involved. This is also lower than a study conducted in Sweden 75%. The possible justification might be due to differences in the study setting, differences in sample size, the and level of the health sector. Another likely explanation may be nurses from these countries possess a higher level of knowledge about preoperative patient education incomparable to the Ethiopian context [33].

In this study, (46.3%) with 95%, CI: (41.1, 51.0) of the participants had a good practice. the result of this study is lower than the study conducted at Ibadan 90% [25] and the Turkish Republic of Northern Cyprus 88.8% [26]. The difference might be no clear job description that nurses can utilize in doing their jobs in the Ethiopian context [20] which results in nurses may not consider preoperative patient education as their responsibility, and the study setting, study unit, and sample size also might be a reason. For example in current study the study unit was only perioperative units and the setting was multicenter which includes five comprehensive specialized hospitals but in the previous study the study population comprises of nurses working in both inpatient and outpatient departments of University College Hospital, Ibadan.

Regarding the determinants of the level of knowledge on preoperative patient education, this study has found that male nurses were found to have good knowledge of preoperative patient education by 2.016 times as compared to females. The reason might be females have an extra workload, most home activities such as bearing and taking care of children cooking, washing etc., are mostly done by females [34]. So due to overload by other additional home activities they might hasn’t enough time to scale up their knowledge.

Those nurses who received training related to preoperative patient education were 3.360 and 2.049 times more likely to have good knowledge and practice of preoperative patient education as compared to counterparts respectively. This finding is supported by the study conducted in Iran [31]. The possible reason might be that training plays an important role in improving the quality of patient care. The need to promote the effectiveness of in-site and off-site training of nurses is an invaluable criterion. Training is necessary to update theoretical and practical knowledge in every aspect of nursing education [35]. Also, this is supported by a study conducted in Hong Cong Moreover, nurses who had participated in surgical or perioperative training courses were detected to have greater satisfaction with preoperative teaching. This can be related to the increasing knowledge about the operation details and the specific perioperative care after attending the relevant surgical training courses and hence they are much eager and more satisfied to use their newly equipped knowledge to teach and provide more preoperative information to patients [18].

On this study also nurses who use guidelines had 1.937 times good knowledge than who do not use it. In the same way in practice, nurses use guidelines had 1.919 times good practice than who do not use it. This is also supported by the qualitative study; the lack of a guideline makes you uneducated and unwilling to give an education (nurse5). This is because the provider of health education requires several skills, which include understanding educational principles; sound, up-to-date, subject-specific knowledge; as well as resources available to support this information [36]. For this reason, I believe that guidelines can change the nurses (educators) knowledge and skill.

The other factors that influenced the patient education practice are the poor knowledge of the nurses. In this finding nurses who had knowledge of preoperative patient education were 3.276 times higher to have a good level of practice. This result is supported by the study conducted in Ibadan, Nigeria [25] and Malaysia [27]. This may be because by gaining new knowledge, their understanding regarding the topic will increase and also can broaden their outlook and give a new point of view thus increasing their self-confidence level. Therefore, they could help more patients in understanding the topic [27].

Another finding is nurses who said no staff shortages in the working hospital were 1.960 times more likely to have good knowledge as compared the nurses who responded with staff shortages. Although I could not find research to support this finding, it might be lack of adequate nurses in the working area did not allow enough time for nurses to read. In addition, nurses who responded that they did not have a lack of staff had 1.846 times better practice than nurses who responded with a lack of staff. This is because nurses’ workload directly influences to the quality of patient care [37]. When the number of staff is less, the workload of nurses will increase, so nurse will not have enough time, so they will not be able to teach.

The present findings revealed how 11 surgical unit nurses in northwest Amhara Comprehensive specialized hospital explore their limitations and barriers to preoperative patient education as well as the facilitator for nurses to teach. Two main themes emerged from the interviews. The first indicates those barriers of preoperative patient education and the second theme facilitators of preoperative patient education.

The result indicates that seven nurses expressed that an organizational factor affects the delivery of preoperative patient education;

All participants described that limitations and inadequacy they encountered in their everyday experience were mainly related to lack of training, lack of guidelines and lack of recognition from the hospital. The finding is in line with Qualitative research conducted in Iran, related to the absence of priority for patient education in the organization and the absence of sufficient specific references are considered as the main factors [38]. The possible justification for this is, that if the organization does not pay attention to it, it will be an obstacle to educatating the patients because there will not have favorable conditions for educating the patients.

The study revealed that, since there is no instruction to teach the patient in the facility, every professional wants to teach his ideas. This is also supported by the study conducted at bonjour lack of educational textbooks, lack of regular plans for patient education were the factors of preoperative patient education [39].

In this study, the low attitude of nurses toward their responsibility was reported to affect preoperative patient education. Some of the participants see the responsible for providing pre-operative education as the responsibility of physicians and anesthesiologists and most of the participant cited a lack of motivation in the patient teaching as a problem. This is also supported by the study conducted, in Hong Kong SAR, China, factors that reportedly influenced the amount of information given to surgical patients were the doctor’s responsibility for providing information (56.3%), and expecting patients to clarify doubts themselves (42.6%) were the factors that were said to prevent nurses from giving complete information to patients [40]. Low levels of attitude among nurses toward their responsibilities may result from a number of things, including the workplace itself, the nurses' backgrounds, their interests in the nursing profession, as well as their families, societies', and consumers' perceptions of the field [41].

Participants also expressed that nurse who has worked for money years teach better in terms of experience. This is true also in a study conducted in Ireland [42] the study explores that experience of the individual nurse influenced the standard of education received by patients. This might be justified as lack of confidence related to inexperience was seen to result in avoidance of engaging in education by nurses, thus omitting the opportunity for patients to ask questions. As nurses’ experience of education of patients before surgery increases; they become familiar with the subject matter thereby improving their knowledge and practice out the procedure.

In this qualitative study, two nurses said that language barriers were among the reasons for the lack of preoperative patient teaching. This defect is similar to the previous study conducted Hong Kong SAR, China [12]. This might be because Participants frequently used face-to-face oral explanations.

Another is that patients attitude toward nurses is declining; they want someone better than nurses. This is supported by a study conducted in Ethiopia, and “the participants expressed negative feelings towards their profession because they feel that the nursing profession is losing the respect of the public”. This is justified as the nurses are not getting respect because there is an over-production of nurses who graduate from the private sector training schools in addition to the government colleges and nursing schools at the universities every year [20].

This qualitative study also found that nurses’ positive interactions with patients are encouraging pre-operative education. Whereas past researchers have found (a study conducted in Hong Cong China) that expecting patients to clarify doubts themselves (42.6%) was the factors that were said to prevent nurses from giving complete information to patients. But many experts believe that the ability to establish accurate communication is one of the most important characteristics of healthcare staff. This results in positive interaction with the patient and facilitates patient teaching [43].

## Conclusion

The knowledge and practice of nurses play an important role in preoperative patient teaching. As shown in this study the knowledge and practice of nurses regarding preoperative patient teaching were found to be inadequate. So, it is better to strengthen training, adequate staffing, equip wards with standardized guidelines and teaching materials, motivate and create a safe working environment helps to provide adequate preoperative teaching. Most nurses explore factors of preoperative patients’ teaching as institutional, Nurse’s related, and patient-related factors.

### Strength and limitation

It is the first study in Ethiopia among nurses regarding this issue and it tried to show their knowledge and practice clearly. One of the limitations of the study is due to the resource constraint; we could not conduct an observational data collection method.

## Data Availability

All data are available upon reasonable request and the readers could contact the corresponding author.

## Abbreviations

AOR: Adjusted Odds Ratio
BSc: Bachelor of Science in Nursing
CI: Confidence Interval
COR: Crude Odds Ratio
Epi- Info: Epidemiological Information
ERAS: Enhanced Recovery After Surgery
ICU: Intensive Care Unit
SPSS: Statistical Package software for Social Sciences
MSc: Master of Science in Nursing
